# Supplementary material for: Stroma-infiltrating T cell spatiotypes define immunotherapy outcomes in adolescent and young adult patients with melanoma
Source: Nat Commun. 2024 Apr 8;15:3014. doi: 10.1038/s41467-024-47301-9 (PMC11002019; doi:10.1038/s41467-024-47301-9)
Supplement: Supplementary file 1 — Supplementary Information [file 41467_2024_47301_MOESM1_ESM.pdf]

Stroma-infiltrating T cell spatiotypes define immunotherapy outcomes in adolescent and young adult patients with melanoma

Xinyu Bai[1,2,3], Grace H. Attrill[1,2,3], Tuba N. Gide[1,2,3], Peter M. Ferguson[1,2,8,9], Kazi J. Nahar[1,2,3], Ping Shang[1,2,3], Ismael A. Vergara[1,2,3], Umaimainthan Palendira[2,3,4], Ines Pires da Silva[1,2,3,7], Matteo S. Carlino[1,7], Alexander M. Menzies[1,2,5,6], Georgina V. Long[*1,2,3,5,6], Richard A. Scolyer[*1,2,3,8,9], James S. Wilmott[*1,2,3], Camelia Quek[*1,2,3]

[1] Melanoma Institute Australia, The University of Sydney, Sydney, NSW, Australia

[2] Faculty of Medicine and Health, The University of Sydney, Sydney, NSW, Australia

[3] Charles Perkins Centre, The University of Sydney, Sydney, NSW, Australia

[4] Centenary Institute, The University of Sydney, Sydney, NSW, Australia

[5] Royal North Shore Hospital, Sydney, NSW, Australia

[6] Mater Hospital, North Sydney, NSW, Australia

[7] Westmead and Blacktown Hospitals, Sydney, NSW, Australia

[8] Royal Prince Alfred Hospital, Sydney, NSW, Australia

[9] NSW Health Pathology, Sydney, NSW, Australia

[*] Contributed equally

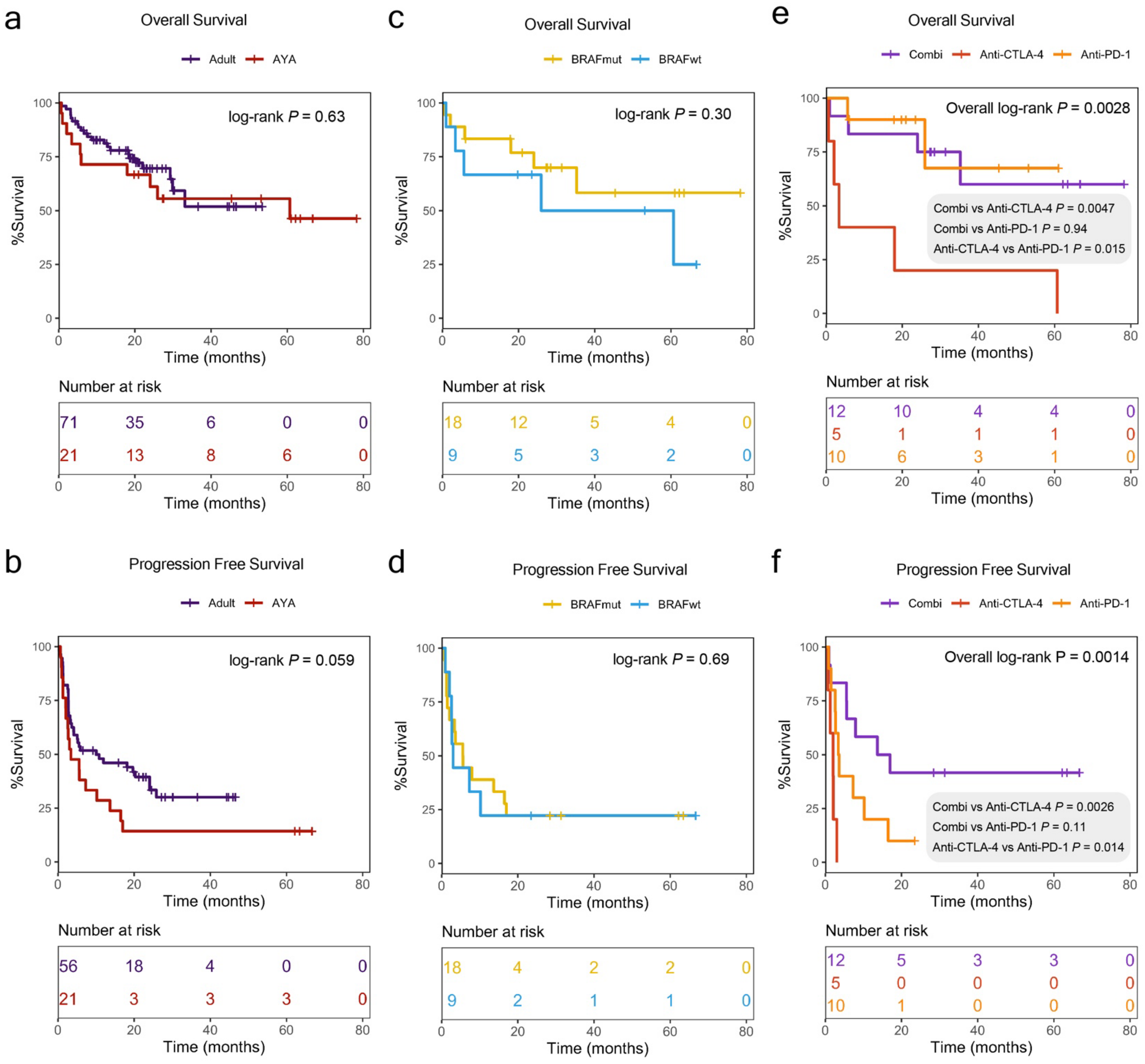

Supplementary Figure 1. Comparisons of overall and progression free survival in patients treated with ICI.
a, b Kaplan-Meier curves comparing the overall and progression-free survival between AYA (n = 21) and adult (n = 71) patients treated with ICI in the advanced setting. **c, d** Kaplan-Meier curves comparing the overall and progression-free survival between AYA patients with (n = 18) and without (n = 9) *BRAF* mutation treated with ICI. **e, f** Kaplan-Meier curves comparing the overall and progression-free survival between AYA patients treated with anti-PD-1 (n = 10), anti-CTLA-4 (n = 5) and combination (Combi, anti-PD-1+anti-CTLA-4; n = 12) ICI.

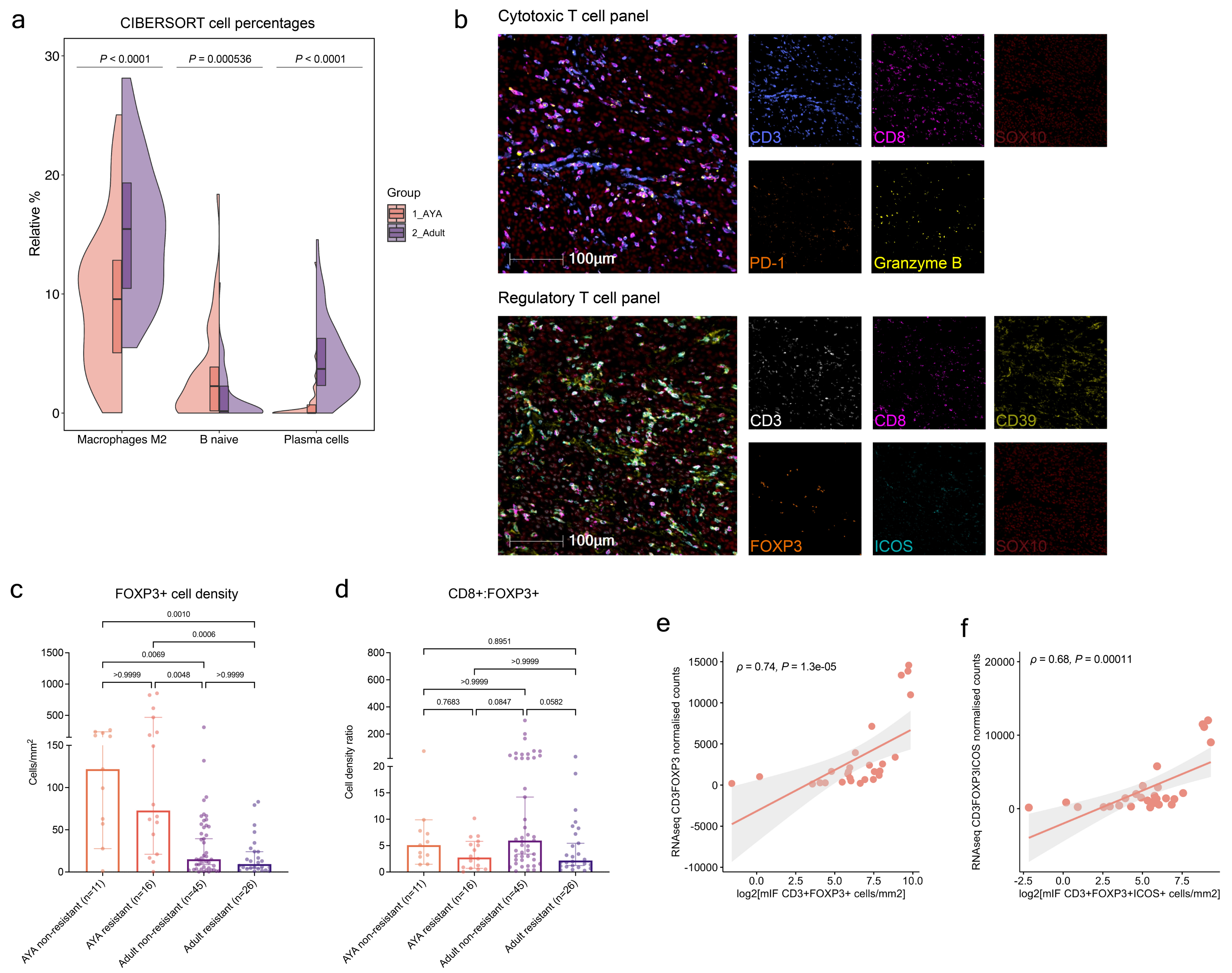

Supplementary Figure 2. Transcriptomic and cellular immune profiles are unique in AYA melanoma patients compared to older adults.

a CIBERSORT immune cell proportion comparisons between AYA (n = 28) and adult (n = 71) melanomas. Violin graph with boxplot shows median and interquartile range of immune cell subsets in AYA and adult melanomas. **b** Representative images of the AYA mIF T cell panels; scale bars represent 50 µm (multiplexed) and 100 µm (single marker). **c** Comparison of $FOXP3^{+}$ cell densities (cells/mm^2) between ICI-resistant and non-resistant AYA and adult patients; bars represent median and 95% CI. **d** Comparison of the CD8:FOXP3 cell ratio between ICI-resistant and non-resistant AYA and adult patients; bars represent median and 95% CI. **e, f** Spearman's correlation between the RNA expressions of *CD3* and *FOXP3* (**e**), or *CD3*, *FOXP3* and *ICOS* (**f**) with the respective mIF protein quantifications in AYA melanoma samples (n = 28).

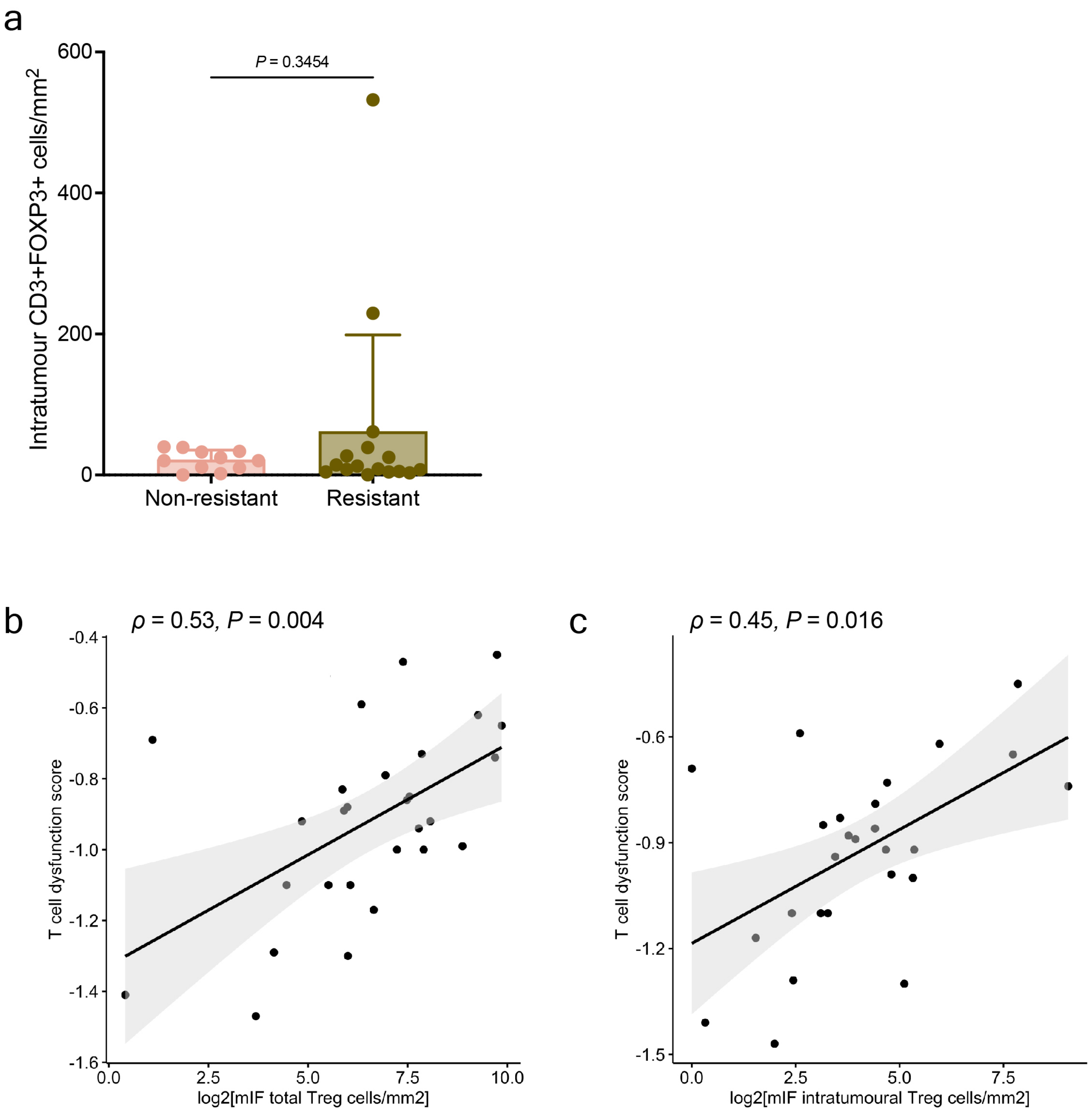

Supplementary Figure 3. Spatial enrichment of T_{regs} is correlated with T cell dysfunction.
a Comparison of intratumoral T_{reg} density between ICI non-resistant and resistant AYA patients (cohort 1); P value of Mann-Whitney test is shown. **b** Spearman's correlation between total T_{reg} density and T cell dysfunction scores of cohort 1 AYA patients. **c** Spearman's correlation between intratumoral T_{reg} density and T cell dysfunction scores of cohort 1 AYA patients.

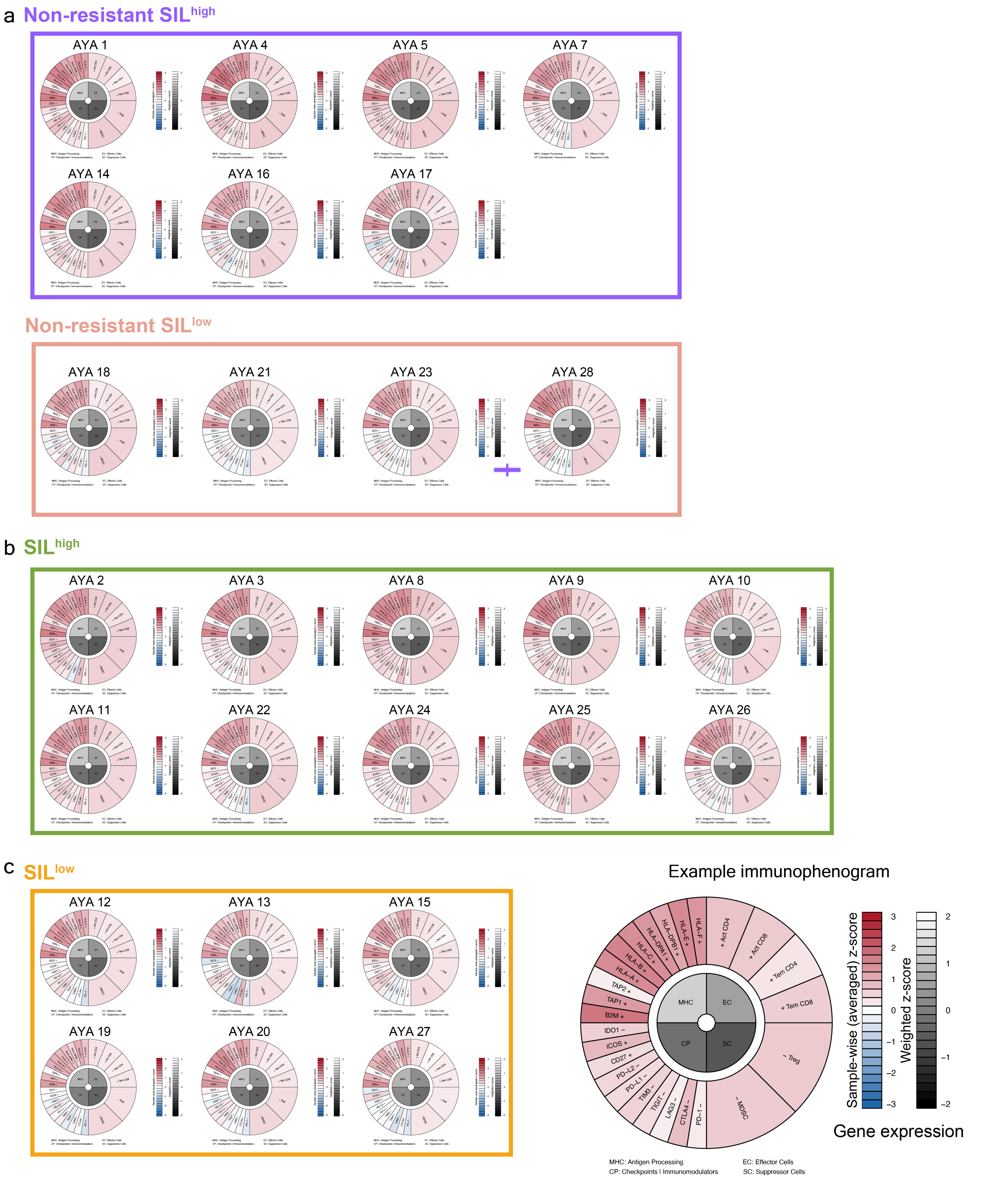

Supplementary Figure 4. Immunophenograms of ICI non-resistant and resistant subtypes of AYA melanoma.
a Immunophenograms of ICI non-resistant patients. **b** Immunophenograms of Group 1 (SIL^{high}) ICI-resistant patients. **c** Immunophenograms of Group 2 (SIL^{low}) ICI-resistant patients. Genes, category acronyms, and colour legends are shown in the example Immunophenogram at the bottom right. The +/- sign after the gene name represents the weighting when calculating the immunophenoscore.

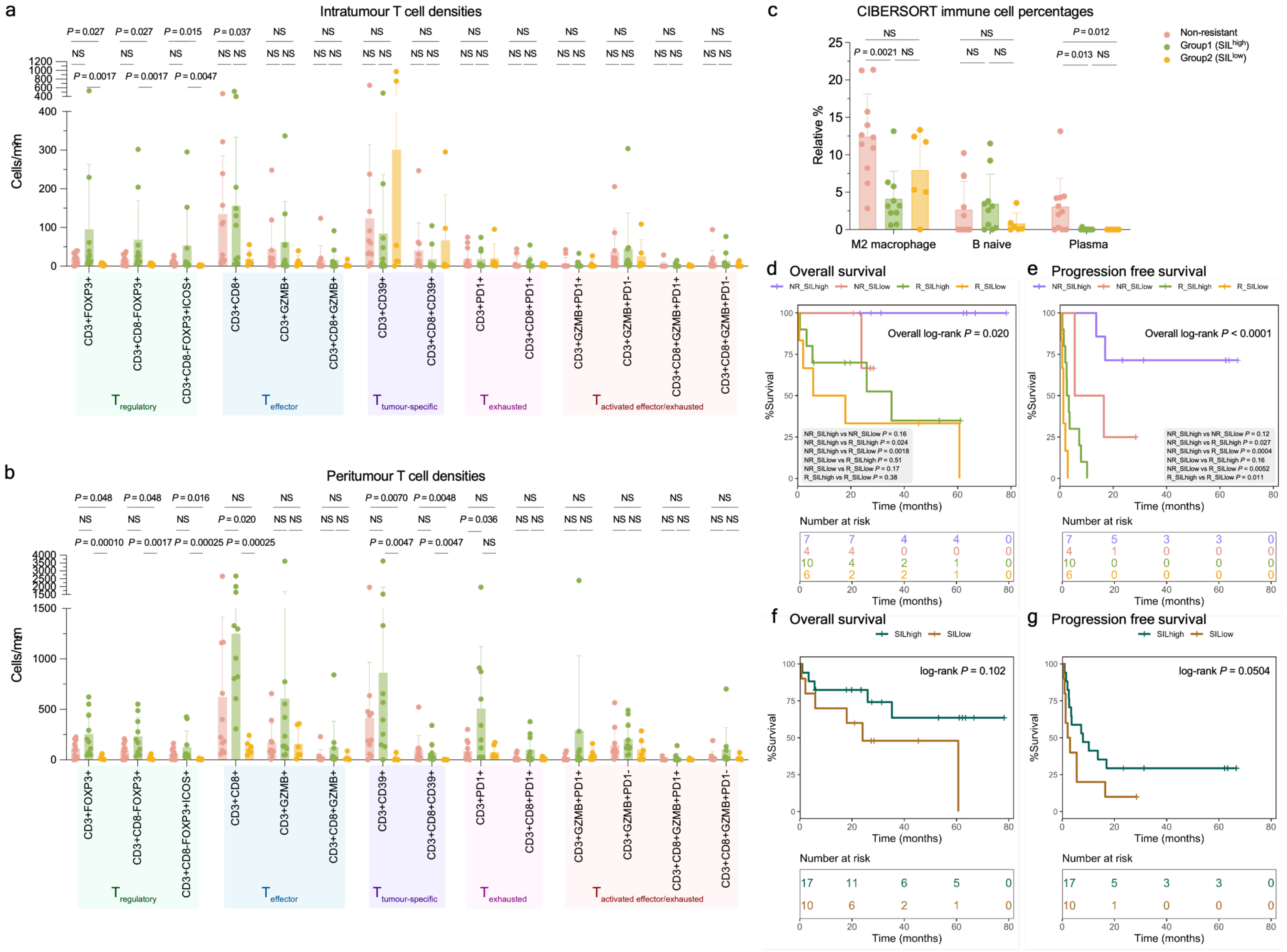

a
Intratumour T cell densities
P = 0.027
P = 0.027
P = 0.015
P = 0.037
P = 0.0017
P = 0.0017
P = 0.0047
NS
Cells/mm²
CD3+FOXP3+
CD3+CD8-FOXP3+
CD3+CD8-FOXP3+ICOS+
CD3+CD8+
CD3+GZMB+
CD3+CD8+GZMB+
CD3+CD39+
CD3+CD8+CD39+
CD3+PD1+
CD3+CD8+PD1+
CD3+GZMB+PD1+
CD3+GZMB+PD1-
CD3+CD8+GZMB+PD1+
CD3+CD8+GZMB+PD1-
T regulatory
T effector
T tumour-specific
T exhausted
T activated effector/exhausted
b
Peritumour T cell densities
P = 0.048
P = 0.048
P = 0.016
P = 0.020
P = 0.0070
P = 0.0048
P = 0.036
P = 0.00010
P = 0.0017
P = 0.00025
P = 0.00025
P = 0.0047
P = 0.0047
c
CIBERSORT immune cell percentages
Non-resistant
Group1 (SIL^high)
Group2 (SIL^low)
Relative %
P = 0.0021
P = 0.012
P = 0.013
M2 macrophage
B naive
Plasma
d
Overall survival
NR_SILhigh
NR_SILlow
R_SILhigh
R_SILlow
Overall log-rank P = 0.020
%Survival
Time (months)
NR_SILhigh vs NR_SILlow P = 0.16
NR_SILhigh vs R_SILhigh P = 0.024
NR_SILhigh vs R_SILlow P = 0.0018
NR_SILlow vs R_SILhigh P = 0.51
NR_SILlow vs R_SILlow P = 0.17
R_SILhigh vs R_SILlow P = 0.38
Number at risk
7 7 4 4 0
4 4 0 0 0
10 4 2 1 0
6 2 2 1 0
e
Progression free survival
Overall log-rank P < 0.0001
NR_SILhigh vs NR_SILlow P = 0.12
NR_SILhigh vs R_SILhigh P = 0.027
NR_SILhigh vs R_SILlow P = 0.0004
NR_SILlow vs R_SILhigh P = 0.16
NR_SILlow vs R_SILlow P = 0.0052
R_SILhigh vs R_SILlow P = 0.011
Number at risk
7 5 3 3 0
4 1 0 0 0
10 0 0 0 0
6 0 0 0 0
f
Overall survival
SILhigh
SILlow
log-rank P = 0.102
Number at risk
17 11 6 5 0
10 6 2 1 0
g
Progression free survival
log-rank P = 0.0504
Number at risk
17 5 3 3 0
10 1 0 0 0

Supplementary Figure 5. Distinct cellular profiles and survival outcomes in ICI non-resistant, SIL^{high} and SIL^{low} subtypes of AYA melanoma.

a Intratumoral T cell density comparisons between ICI non-resistant and resistant Group 1 (SIL^{high}) and Group 2 (SIL^{low}) AYA patients; bar and lines represent mean and standard deviation; P values of Mann-Whitney tests are shown; NS, not significant (P > 0.05). **b** Peritumoral T cell density comparisons between ICI non-resistant and resistant Group 1 (SIL^{high}) and Group 2 (SIL^{low}) AYA patients; bar and lines represent mean and standard deviation; P values of Mann-Whitney tests are shown; NS, not significant (P > 0.05). **c** Comparisons of CIBERSORT immune cell proportions between ICI non-resistant and resistant Group 1 and Group 2 AYA patients; bar and lines represent mean and standard deviation; P values of Mann-Whitney tests are shown; NS, not significant (P > 0.05). **d, e** Kaplan-Meier curves comparing the overall and progression-free survival between SIL^{high} and SIL^{low} subgroups of ICI resistant and non-resistant AYA patients. **f, g** Kaplan-Meier curves comparing the overall and progression-free survival between SIL^{high} and SIL^{low} groups of all AYA patients with recorded outcome.

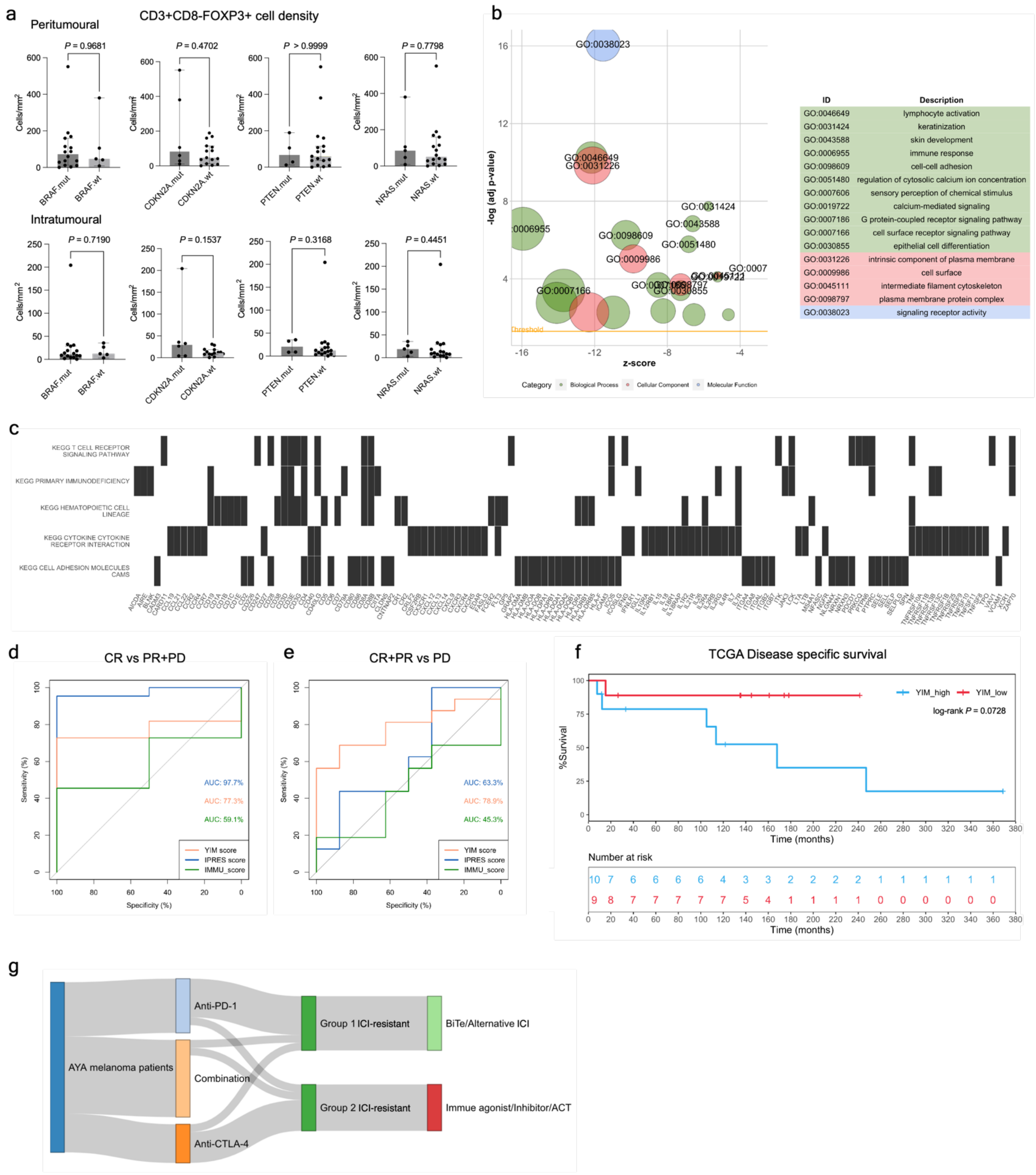

Supplementary Figure 6. Differentially expressed genes in AYA patients can help to predict response and inform treatment strategies in ICI-resistant patients.

a Comparison of peritumor T_{reg} cell densities in AYA melanomas with and without the respective somatic mutations; bar plot shows mean and standard deviation. **b** Overrepresentation analysis of Group 1 versus Group 2 differentially expressed genes (DEGs) with GO pathway datasets, size of the circles represents gene count. **c** DEGs mapped to the significantly enriched KEGG gene sets, upregulated in Group 1 (SIL^{high}) and downregulated in Group 2 (SIL^{low}) AYA immunotherapy resistant melanomas. **d** Receiver operating characteristic curve of Young Immunosuppressive Melanoma (YIM) scores for the prediction of ICI outcome in AYA complete responders (CR) versus partial responders (PR) and progressive disease (PD) patients. **e** Receiver operating characteristic curve of YIM scores for the prediction of ICI outcome in AYA CR and PR versus PD patients. **f** Kaplan-Meier curves comparing the disease specific survival between YIM-high and YIM-low groups of TCGA AYA patients (n = 19). **g** Model treatment switch plan for ICI-resistant patients; bi-specific T cell engager (BiTE); adoptive cell transfer (ACT).

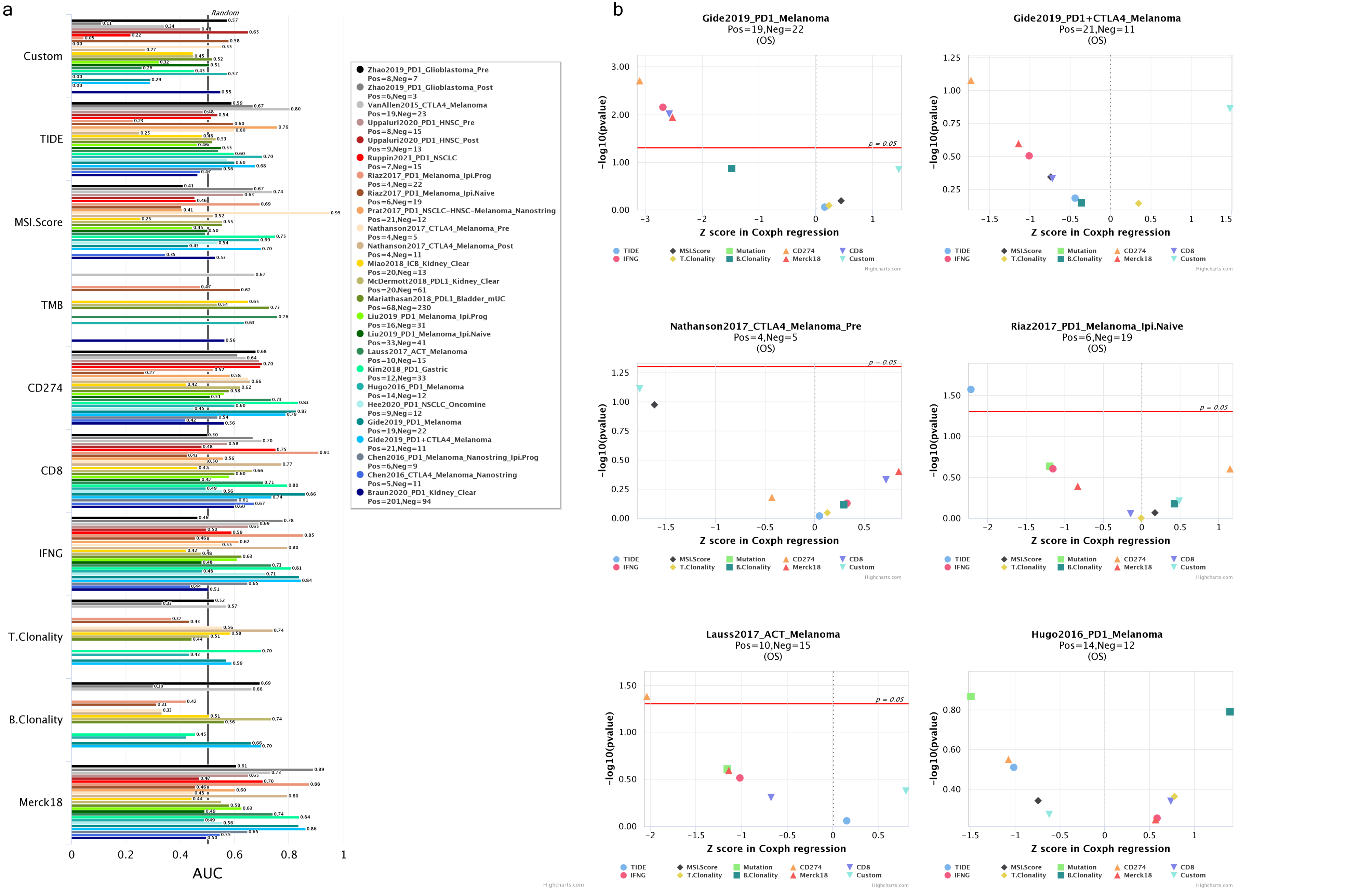

Supplementary Figure 7. YIM score does not predict ICI response in adult cancer patients.

a Biomarker evaluation scores calculated with the Tumor Immune Dysfunction and Exclusion (TIDE) computational model comparing the YIM gene set (shown as “Custom”) identified from this study with other published signatures. **b** Survival prediction for published cohorts using YIM and published signatures, plots show the Cox proportional-hazards regression z score and p values.

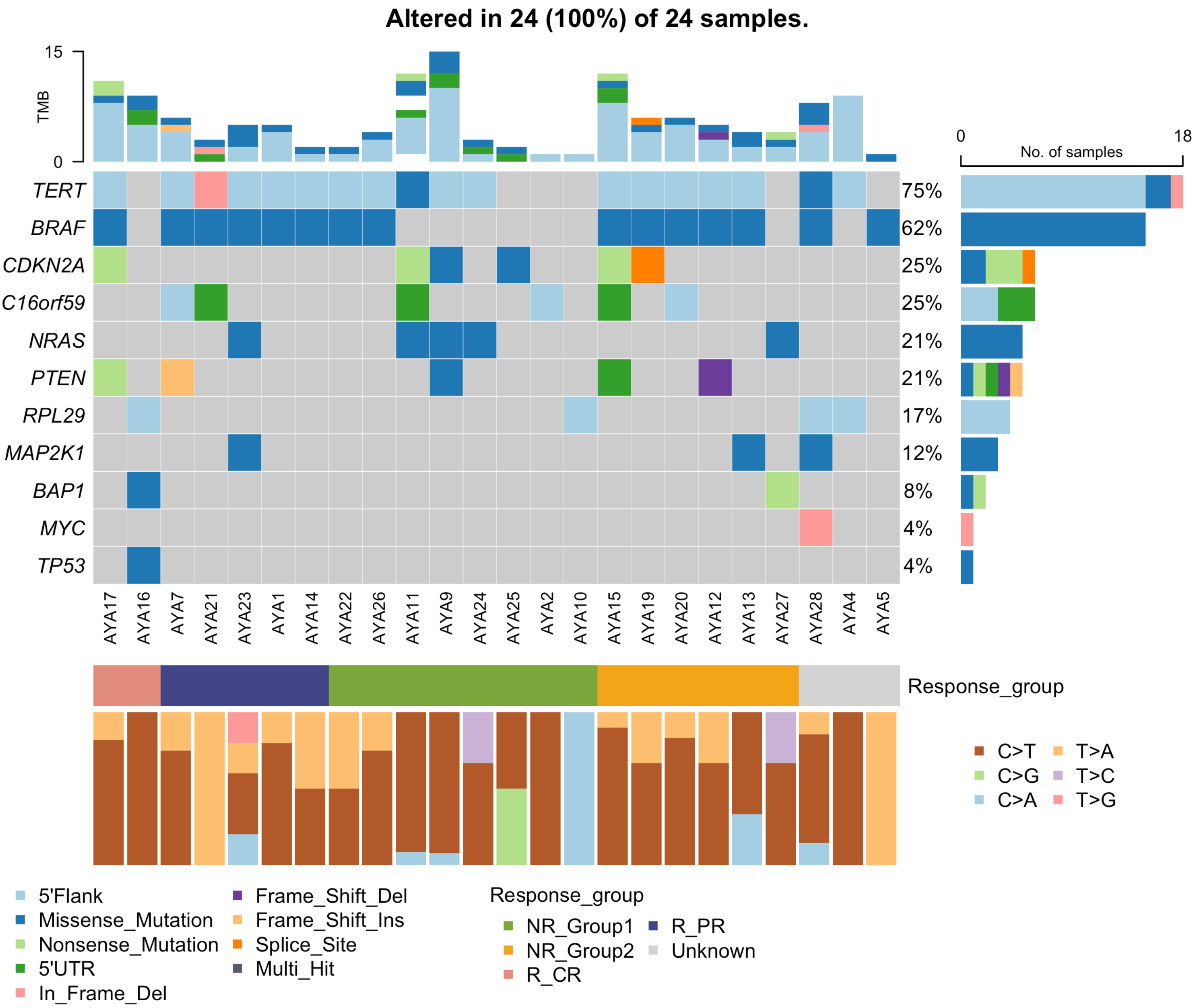

Supplementary Figure 8. List of somatic variants in ICI-treated AYA melanoma patients.

Oncoprint plot showing the types of somatic variants in cohort 1 (MIA) AYA patients. The variant types include 5 prime flank region, missense mutation, nonsense mutation, 5 prime untranslated region, in frame deletion, frame shift deletion, frame shift insersion, and splice site mutation. The response groups include non-responder (NR) Group 1 (SIL^{high}), NR Group 2 (SIL^{low}), complete responder (R_CR) and partial responder (R_PR).
