# Supplementary material for: Stroma-infiltrating T cell spatiotypes define immunotherapy outcomes in adolescent and young adult patients with melanoma
Source: Nat Commun. 2024 Apr 8;15:3014. doi: 10.1038/s41467-024-47301-9 (PMC11002019; doi:10.1038/s41467-024-47301-9)
Supplement: Supplementary file 3 — Description of Additional Supplementary Files [file 41467_2024_47301_MOESM3_ESM.pdf]

Description of Additional Supplementary Files

Supplementary Data 1

Description: Patient characteristics.

Supplementary Data 2

Description: AYA and adult cohort comparisons.

Supplementary Data 3

Description: AYA vs adult CIBERSORT immune cell proportions.

Supplementary Data 4

Description: Cell-type specific genes.

Supplementary Data 5

Description: AYA Group 1 vs Group 2 mIF cell density analysis.

Supplementary Data 6

Description: Group 1 vs Group 2 differentially expressed genes.

Supplementary Data 7

Description: Enriched KEGG and GO genesets between AYA immunotherapy resistant subgroups (Group 1 vs Group 2).

Supplementary Data 8

Description: Predictive gene signatures of immunotherapy response and Singscores of AYA patients.

Supplementary Data 9

Description: Enriched KEGG and GO genesets in YIM signature.

Supplementary Data 10

Description: DNA targeted sequencing panel.

Supplementary Data 11

Description: Cohort 1 (AYA n=28) somatic variants.

Supplementary Data 12

Description: Drug targets and expression in AYA PD patients.

Supplementary Data 13

Description: AYA mIF panels and antibody details.
