# Supplementary material for: Stroma-infiltrating T cell spatiotypes define immunotherapy outcomes in adolescent and young adult patients with melanoma
Source: Nat Commun. 2024 Apr 8;15:3014. doi: 10.1038/s41467-024-47301-9 (PMC11002019; doi:10.1038/s41467-024-47301-9)
Supplement: Supplementary file 5 — Reporting Summary [file 41467_2024_47301_MOESM5_ESM.pdf]

nature portfolio

Corresponding author(s): Camelia Quek

Last updated by author(s): Feb 22, 2024

Reporting Summary

Nature Portfolio wishes to improve the reproducibility of the work that we publish. This form provides structure for consistency and transparency in reporting. For further information on Nature Portfolio policies, see our Editorial Policies and the Editorial Policy Checklist.

Statistics

For all statistical analyses, confirm that the following items are present in the figure legend, table legend, main text, or Methods section.

n/a	Confirmed	
☐	☒	The exact sample size (*n*) for each experimental group/condition, given as a discrete number and unit of measurement
☐	☒	A statement on whether measurements were taken from distinct samples or whether the same sample was measured repeatedly
☐	☒	The statistical test(s) used AND whether they are one- or two-sided<br>*Only common tests should be described solely by name; describe more complex techniques in the Methods section.*
☐	☒	A description of all covariates tested
☐	☒	A description of any assumptions or corrections, such as tests of normality and adjustment for multiple comparisons
☐	☒	A full description of the statistical parameters including central tendency (e.g. means) or other basic estimates (e.g. regression coefficient) AND variation (e.g. standard deviation) or associated estimates of uncertainty (e.g. confidence intervals)
☐	☒	For null hypothesis testing, the test statistic (e.g. *F*, *t*, *r*) with confidence intervals, effect sizes, degrees of freedom and *P* value noted<br>*Give P values as exact values whenever suitable.*
☒	☐	For Bayesian analysis, information on the choice of priors and Markov chain Monte Carlo settings
☐	☒	For hierarchical and complex designs, identification of the appropriate level for tests and full reporting of outcomes
☐	☒	Estimates of effect sizes (e.g. Cohen's *d*, Pearson's *r*), indicating how they were calculated

Our web collection on statistics for biologists contains articles on many of the points above.

Software and code

Policy information about availability of computer code

Data collection	Data were collected manually for individual patients from MRD2 (melanoma research database v2) software of the Melanoma Institute Australia
Data analysis	The following analysis tools was used: (1) DNA sequencing analysis (VariantPlex Archer Analysis Pipeline v5.1.8, Cancer Genome Interpreter and maftools v2.8.05), (2) RNA sequencing analysis (Trimmomatic v3.0, TopHat v2.0.8, Bowtie v2.1.0, SAMtools v0.1.19, FastQC, RNA-SeQC, HTSeq, DESeq2 v1.32, CIBERSORT, Immunophenogram, MSigDB, clusterProfiler v4.0.5, Singscore, DGIdb, networkD3), (3) Multiplexed immunofluorescence image analysis (inForm v2.4.2 and HALO v3.2) and (4) Statistical analysis (R v4.1, pROC R packages and GraphPad Prism 9). Further details of the parameters and algorithms were described in the methods.

For manuscripts utilizing custom algorithms or software that are central to the research but not yet described in published literature, software must be made available to editors and reviewers. We strongly encourage code deposition in a community repository (e.g. GitHub). See the Nature Portfolio guidelines for submitting code & software for further information.

Data

Policy information about availability of data

All manuscripts must include a data availability statement. This statement should provide the following information, where applicable:

- Accession codes, unique identifiers, or web links for publicly available datasets
- A description of any restrictions on data availability
- For clinical datasets or third party data, please ensure that the statement adheres to our policy

The authors declare that the RNA sequencing data for AYA patients has been deposited in the European Nucleotide Archive under accession number PRJEB52880. Future details are included in the Data Availability section of the manuscript. Information related to databases/datasets generated and/or used in the study are mentioned in the Data Availability section of the manuscript. All other data supporting the findings of this study are available upon reasonable request from the corresponding authors.

Research involving human participants, their data, or biological material

Policy information about studies with human participants or human data. See also policy information about sex, gender (identity/presentation), and sexual orientation and race, ethnicity and racism.

Reporting on sex and gender	For clinicopathological characteristics, we used gender for Fisher-exact test as outline in Table 2 and methods.
Reporting on race, ethnicity, or other socially relevant groupings	Not applicable.
Population characteristics	Supplementary Table 1 provided the details of the patient characteristics and further outlined the details in the methods.
Recruitment	The criteria as outlined in the methods.
Ethics oversight	The research protocol was approved by the institutional review boards and ethics committees (Sydney Local Health District Human Research Ethics Committee, Protocol No. X-15-0454 and HREC/11/RPAH/444).

Note that full information on the approval of the study protocol must also be provided in the manuscript.

Field-specific reporting

Please select the one below that is the best fit for your research. If you are not sure, read the appropriate sections before making your selection.

[x] Life sciences [ ] Behavioural & social sciences [ ] Ecological, evolutionary & environmental sciences

For a reference copy of the document with all sections, see nature.com/documents/nr-reporting-summary-flat.pdf

Life sciences study design

All studies must disclose on these points even when the disclosure is negative.

Sample size	Biospecimens retrieved from AYA melanoma patients treated with immunotherapy are very rare, so we aimed to use all samples in the Biospecimen Bank and Research Database in Melanoma Institute Australia. To validate our findings with an independent study, we analyzed sequencing data retrieved from TCGA-SKCM as outlined in the methods.
Data exclusions	As stated in the extended data, 4 patients (AYA 3, AYA6, AYA8, AYA18) from this cohort was removed from somatic DNA variant analysis due to poor tumor purity.
Replication	All experiments were performed independently for each sample (n=28).
Randomization	Our work did not include any intervention, so randomization was not applicable.
Blinding	Our work did not include any intervention, so blinding was not applicable.

Reporting for specific materials, systems and methods

We require information from authors about some types of materials, experimental systems and methods used in many studies. Here, indicate whether each material, system or method listed is relevant to your study. If you are not sure if a list item applies to your research, read the appropriate section before selecting a response.

Materials & experimental systems

n/a	Involved in the study
☐	☒ Antibodies
☒	☐ Eukaryotic cell lines
☒	☐ Palaeontology and archaeology
☒	☐ Animals and other organisms
☐	☒ Clinical data
☒	☐ Dual use research of concern
☒	☐ Plants

Methods

n/a	Involved in the study
☒	☐ ChIP-seq
☒	☐ Flow cytometry
☒	☐ MRI-based neuroimaging

Antibodies

Antibodies used

All antibodies used in this study are outlined in Supplementary Data 13.

Validation

Panel 1 Tregs
Order Antibody Species Clone Catalog # Manufacturer Dilution OPAL (1:100) Secondary Seconday Catalog # Secondary Manufacturer
1 FoxP3 Mouse 236A1E7 ab20034 Abcam 1:250 540 MACH 3 Mouse HRP-Polymer Detection M3M530 Biocare Medical
2 ICOS Rabbit D1K2T 89601 Cell Signalling Technology 1:2000 620 Opal Anti-Ms + Rb HRP NEL821001KT Akoya Biosciences
3 CD8 Mouse C8/144B GA62361-2 Agilent Dako 1:1500 650 Opal Anti-Ms + Rb HRP NEL821001KT Akoya Biosciences
4 CD3 Rabbit MRQ-39 103S Sigma-Aldrich 1:1500 570 Opal Anti-Ms + Rb HRP NEL821001KT Akoya Biosciences
5 CD39 Rabbit EPR20627 ab223842 Abcam 1:1000 520 Opal Anti-Ms + Rb HRP NEL821001KT Akoya Biosciences
6 SOX10 Mouse BC34 3099 Biocare Medical 1:200 690 Opal Anti-Ms + Rb HRP NEL821001KT Akoya Biosciences

Panel 2 CD8-T
Order Antibody Species Clone Catalog # Manufacturer Dilution OPAL (1:100) Secondary Seconday Catalog # Secondary Manufacturer
1 Granzyme B Mouse GRB-7 M723501-2 Agilent Dako 1:200 520 MACH 3 Mouse HRP-Polymer Detection M3M530 Biocare Medical
2 PD-1 Rabbit EPR4877(2) ab137132 Abcam 1:1500 540 Opal Anti-Ms + Rb HRP NEL821001KT Akoya Biosciences
3 CD8 Mounse C8/144B GA62361-2 Agilent Dako 1:1500 650 Opal Anti-Ms + Rb HRP NEL821001KT Akoya Biosciences
4 CD3 Rabbit MRQ-39 103S Sigma-Aldrich 1:1500 570 Opal Anti-Ms + Rb HRP NEL821001KT Akoya Biosciences
5 SOX10 Mounse BC34 3099 Biocare Medical 1:200 690 Opal Anti-Ms + Rb HRP NEL821001KT Akoya Biosciences

Clinical data

Policy information about clinical studies
All manuscripts should comply with the ICMJEguidelines for publication of clinical research and a completedCONSORT checklist must be included with all submissions.

Clinical trial registration

ACTRN12613000385741, NCT01844505, NCT01866319, NCT02089685, NCT02362594, NCT02374242, NCT03068455, NCT03980314

Study protocol

The protocols have been published for individual trials at the time of publication.

Data collection

Data were collected by the principal investigators of the trials.

Outcomes

The outcomes and patients' trial status are outlined in Methods and Supplementary Data 1.
